# Supplementary figures and images for: Proteomic analysis defines the interactome of telomerase in the protozoan parasite, Trypanosoma brucei
Source: Front Cell Dev Biol. 2023 Mar 16;11:1110423. doi: 10.3389/fcell.2023.1110423 (PMC10061497; doi:10.3389/fcell.2023.1110423)

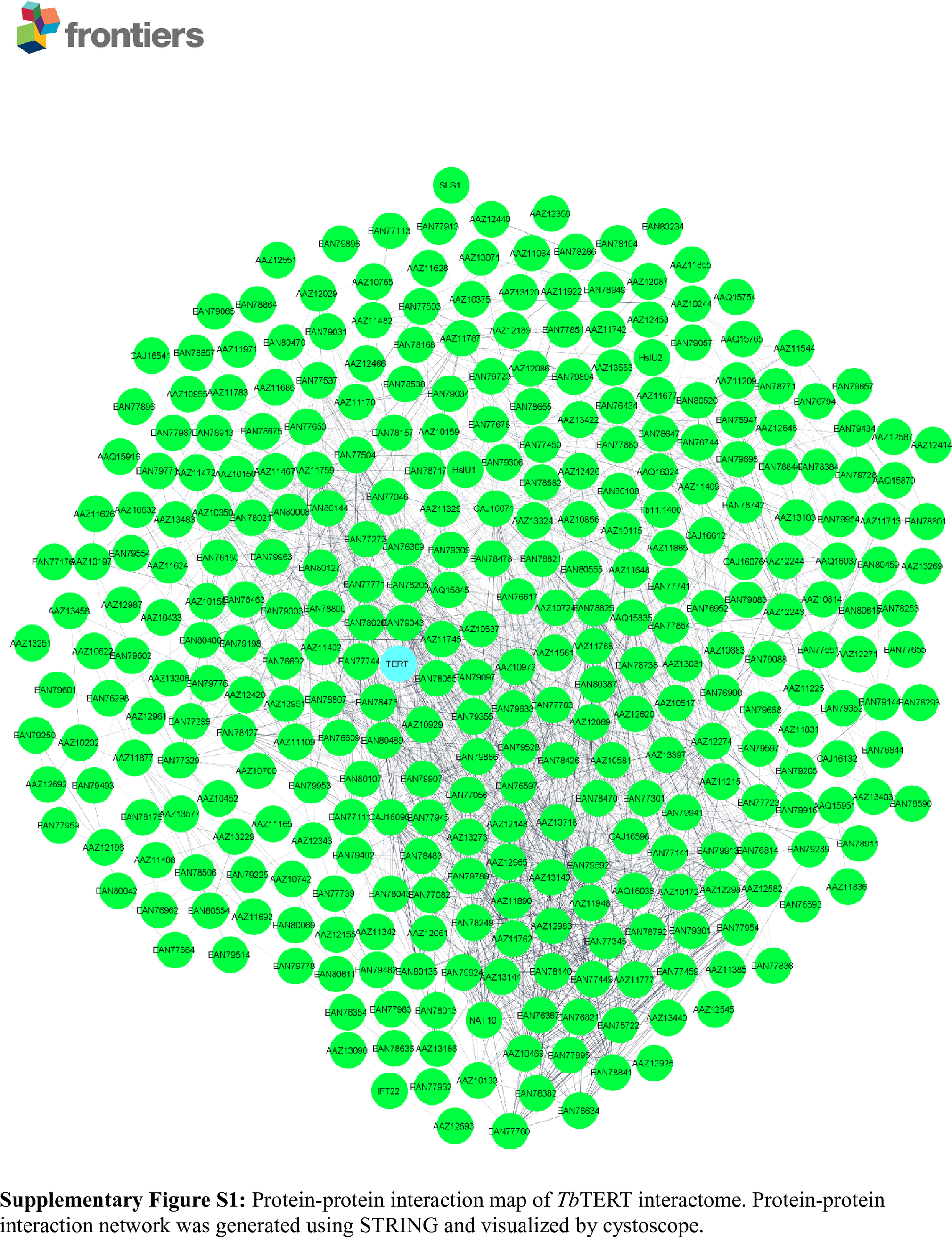

Supplement: Supplementary file 2 [file Image1.tif]
